# EEG-Based Emotion Classification Using a Deep Neural Network and Sparse Autoencoder

**DOI:** 10.3389/fnsys.2020.00043

**Published:** 2020-09-02

**Authors:** Junxiu Liu, Guopei Wu, Yuling Luo, Senhui Qiu, Su Yang, Wei Li, Yifei Bi

**Affiliations:** ^1^School of Electronic Engineering, Guangxi Normal University, Guilin, China; ^2^Guangxi Key Lab of Multi-Source Information Mining & Security, Guangxi Normal University, Guilin, China; ^3^Guangxi Key Laboratory of Wireless Wideband Communication and Signal Processing, Guilin, China; ^4^Department of Computer Science and Software Engineering, Xi'an Jiaotong-Liverpool University, Suzhou, China; ^5^Academy for Engineering & Technology, Fudan University, Shanghai, China; ^6^Department of Electronic Engineering, The University of York, York, United Kingdom; ^7^College of Foreign Languages, University of Shanghai for Science and Technology, Shanghai, China; ^8^Department of Psychology, The University of York, York, United Kingdom

**Keywords:** EEG, emotion recognition, convolutional neural network, sparse autoencoder, deep neural network

## Abstract

Emotion classification based on brain–computer interface (BCI) systems is an appealing research topic. Recently, deep learning has been employed for the emotion classifications of BCI systems and compared to traditional classification methods improved results have been obtained. In this paper, a novel deep neural network is proposed for emotion classification using EEG systems, which combines the Convolutional Neural Network (CNN), Sparse Autoencoder (SAE), and Deep Neural Network (DNN) together. In the proposed network, the features extracted by the CNN are first sent to SAE for encoding and decoding. Then the data with reduced redundancy are used as the input features of a DNN for classification task. The public datasets of DEAP and SEED are used for testing. Experimental results show that the proposed network is more effective than conventional CNN methods on the emotion recognitions. For the DEAP dataset, the highest recognition accuracies of 89.49% and 92.86% are achieved for valence and arousal, respectively. For the SEED dataset, however, the best recognition accuracy reaches 96.77%. By combining the CNN, SAE, and DNN and training them separately, the proposed network is shown as an efficient method with a faster convergence than the conventional CNN.

## 1. Introduction

The Brain–Computer Interface (BCI) directly connects human (or animal) brain activity with artificial effectors (Kübler et al., 2009), which provides an interactive pathway between the human brain and external devices for various applications. The process of such an interaction starts by recording the brain activity through the signal processing and analysis to detect the users' intent (Tabar and Halici, 2016). BCI systems and their various implementations have been subjects of ongoing study for decades, and one of the most appealing research directions is emotion recognition due to its potential applications in numerous scenarios. Both non-physiological and physiological signals could be employed for emotion detections. Non-physiological signals include facial expression images (Lane et al., 1997), voice signals (Scherer, 1995), and body gesture (Cheng and Liu, 2008). Compared to the non-physiological signals, physiological signals can be detected by some wearable devices, such as an electroencephalogram (EEG) (Zheng, 2017), electromyogram (Hiraiwa et al., 1989), electrocardiogram (Agrafioti et al., 2012), the galvanic skin response, blood volume pressure, and a photoplethysmogram. Among these physiological signals, EEG signals have been widely used for research into emotion recognition (Chi et al., 2012; Huang et al., 2015; Li et al., 2016; Liu et al., 2018c). Captured from the scalp by a number of EEG electrodes, emotion could be reflected immediately by an EEG signal once a subject receives the stimulations.

There are two conventional rules to follow when categorizing human emotions, namely, the discrete basic emotion description and the dimension approaches. According to the discrete basic emotion description approach, emotions can be classified into six basic emotions: sadness, joy, surprise, anger, disgust, and fear (van den Broek, 2013). For the dimension approach, the emotions can be classified into two (valence and arousal) or three dimensions (valence, arousal, and dominance) (Zheng and Lu, 2015). Among these dimensions, valence describes the level of positivity or negativity of one person, and arousal describes the level of excitement or apathy of emotion. The scale of dominance ranges from submissive (without control) to dominance (empowered). The emotion recognition is usually based on the dimension approach because of its simplicity compared to the discrete basic emotion description (Zheng and Lu, 2015).

Early works on emotion recognition through analysing EEG signal could be traced back to more than 50 years ago (Fink, 1969). Many new methods on feature extraction and classification have recently been proposed for emotion detection (Petrantonakis and Hadjileontiadis, 2010). For the feature extraction, two types of feature are commonly used to analyze EEG signals: time-domain and frequency-domain features. Time-domain features capture the temporal information of signals, such as the fractal dimension (Hjorth, 1970), Hjorth, and higher-order crossing features (Petrantonakis and Hadjileontiadis, 2010). The frequency-domain features can extract the useful information from the frequency perspective under different frequency bands. For instance, the EEG signal could be decomposed into δ (1–3 Hz), θ (4–7 Hz), α (8–13 Hz), β (14–30 Hz), and γ bands (31–50 Hz) (Hjorth, 1970; Li and Lu, 2009; Petrantonakis and Hadjileontiadis, 2010; Nie et al., 2011), where the features can be extracted from each of them. In addition, other features, such as Deep Forest (Zhou and Feng, 2017), Statistical Characteristics (SC), Differential Entropy (DE) feature (Zheng et al., 2014), Pearson Correlation Coefficient (PCC) feature (Lewis et al., 2007), and Principal Component Analysis (PCA) (Subasi and Gursoy, 2010), are also used in emotion recognitions.

In the meantime, various classification methods have been used for emotion recognition, such as k-Nearest Neighbor (Bahari and Janghorbani, 2013), Multi-Layer Perceptron (Orhan et al., 2011). A Support Vector Machine (SVM) and Linear Regression (LR) were used in Wang et al. (2019), but recognition accuracy can be improved. In recent years, deep neural networks (DNN) (Tripathi et al., 2017) has been developed into one of the most effective and popular methods in many research fields (Fu et al., 2017; Liu et al., 2018a,b, 2019; Luo et al., 2018). Convolutional Neural Networks (CNN) are widely used in computer vision, image classifications, visual tracking (Danelljan et al., 2016), segmentation, and object detections (Girshick et al., 2014). EEG emotion classification using the CNN method was also explored in the approaches of Tripathi et al. (2017). Cascade and parallel convolutional recurrent neural networks have been used for EEG human-intended movements classification tasks (Zhang et al., 2018). Additionally, before applying the CNN, EEG data could be converted to image representation after feature extraction (Tabar and Halici, 2016). However, the accuracy of emotion recognition by using only CNN is not high. In the work of Zhang et al. (2017), a deep learning framework consisting of the sparse autoencoder (SAE) and logistic regression was used to classify EEG emotion status. The sparse autoencoder was employed for feature extraction, and logistic regression was used to predict affective states. The SAE is an unsupervised machine learning algorithm. By calculating the error between the output of the SAE and original input, data could be reconstructed and useful features could be extracted for classification task. However, accuracy of that work is not high and there are no experiments for comparing to verify the work of the SAE.

**Figure 1:**
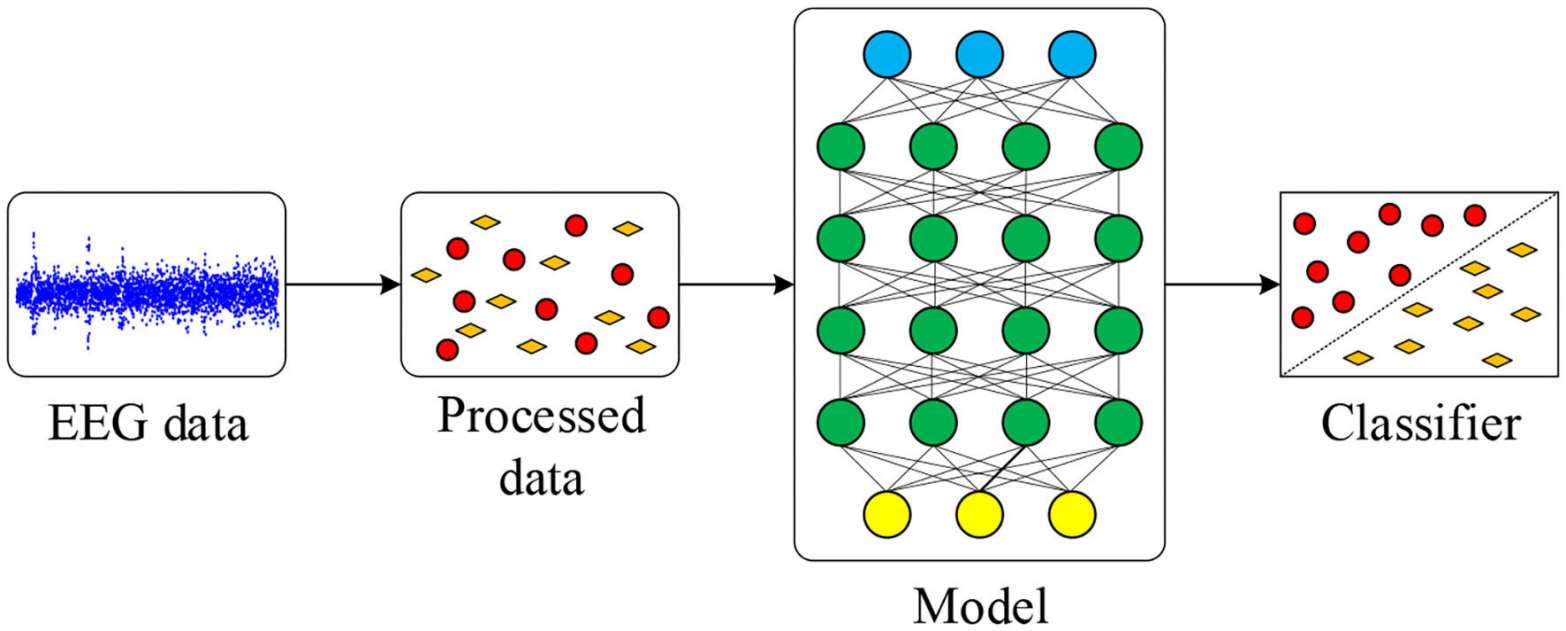
Emotion classification procedure in this work.

In this work, a novel network model combining the CNN, SAE, and DNN to convert EEG time series into 2D images for a good emotion classification performance is proposed. The EEG signal is decomposed into several different bands. Based on frequency, time, and location information, the 2D features are extracted from EEG data. Then convolutional layers of the CNN are trained and used for further extracting features. The SAE is used for reconstructing data obtained from convolutional layers, and the DNN is used for classification. Compared to other approaches, the proposed neural network model, which leverages the benefits of convolutional layers of the CNN and sparsity of the SAE, demonstrates a good classification accuracy and fast convergence. The procedure of the proposed method is summarized in Figure 1. Original EEG data are pre-processed, and features are extracted for deep learning model. After training and testing on the model, final classification results are obtained.

The rest of this paper is organized as follows: the proposed neural network model is presented in section 2. Datasets and experimental results are provided in section 3. Section 4 summaries the work and discusses the future work.

## 2. Deep Learning Framework

In this section, fundamental principles and essential network modules are presented. The novel model is also introduced in detail.

### 2.1. Convolution Neural Network (CNN)

The features extracted from original EEG data are sent to the CNN first. The CNN model includes several convolution-pooling layer pairs and one output layer. Before sending to the CNN, features are concatenated into image form which is then convolved with several one-dimensional filters in convolution layers. After the pooling layer, the data are further subsampled to images with smaller size. Network weights and filters in the convolution layers are learned through back-propagation algorithm.

In our experiments, data extracted from EEG signal are from four typical frequency bands, which include α (1–7 Hz), β (8–13 Hz), θ (14–30 Hz), and γ bands (30–45 Hz), using a Butterworth band-pass filter. After that, data are reformed into two-dimensional features, such as PCC, which are the input for CNN. Detailed methods of this procedure is presented in sections 3.3 and 3.4. It is worth noting that the two-dimensional features contain not only the frequency but also spatial location information of each electrode (Tabar and Halici, 2016). To preserve this information, one-dimensional filtering is applied in this work instead of two-dimensional filtering.

The CNN structure is relatively straightforward. Input vector is two-dimensional feature, which can be given by

(1) $$\begin{array}{r} x=\left(\begin{array}{cccc} x_{11} & x_{12} & \ldots & x_{1n}\\ x_{21} & x_{22} & \ldots & x_{2n}\\ \ldots & \ldots & \ldots & \ldots \\ x_{m1} & x_{m2} & \ldots & x_{mn} \end{array}\right), \end{array}$$

where *m* × *n* is the shape of input vector *x*. The input two-dimensional feature is convolved with filters *W*_*k*_ at the convolution layer, which is given by

(2) $$\begin{array}{r} W_{k}=\left(\begin{array}{c} W_{11}\\ W_{21}\\ \ldots \\ W_{i1} \end{array}\right), \end{array}$$

where *i* is length of *W*_*k*_ and *i*<*m* in Equation (1). After the image convolution, output map is formed and the feature map at the given layer is obtained by

(3) $$\begin{array}{r} f\left(\alpha \right)=f\left(W_{k}\times x+b_{k}\right), \end{array}$$

where *W*_*k*_ ∈ *R*^*i* × 1^ is the weight matrix and *b*_*k*_ is the bias value, *k* denotes the filter, for *k* = 1, 2, …, *n* and *n* denotes the total number of filtering in convolutional layer. The activation function is *f*, which is a rectified linear unit (*ReLU*) function in this work. Compared with the traditional neural network activation functions, such as *sigmoid* and *tanh*, *ReLU* is more efficient in avoiding gradient disappearance. *ReLU* function is defined by

(4) $$\begin{array}{r} f\left(\alpha \right)=ReLU\left(\alpha \right)=\ln\;\left(1+e^{\alpha }\right), \end{array}$$

where α is defined in Equation (3). At the max-pooling layer, the feature map is down sampled through the max-pooling function. Max-pooling is used because it is found that the maximum value from the selected values of a given feature map could be effectively extracted using this function.

After the last pooling layer, a fully connected layer follows in which output data from pooling layer is flattened. After that, fully connected layers named DNN are followed. In DNN, the activation function of each layer is also *ReLU*. For the output layer, because there are two classification tasks, including binary classification and multi-class classification, *sigmoid* and *softmax* are used, respectively. For the binary-classification task, *Adadelta* is used as an optimizer, and loss is calculated by binary crossentropy, which is given by

(5) $$\begin{array}{r} loss=-\overset{N}{\underset{n=1}{\sum }}{ŷ}_{i}logy_{i}+\left(1-{ŷ}_{i}\right)log\left(1-{ŷ}_{i}\right), \end{array}$$

where *N* is number of samples, *y*_*i*_ is the value, which is a form of one-hot code, and $\hat{y_{i}}$ is the output from the output layer where *sigmoid* is used. For multi-class classification, such as three-class classification, *Adam* is used as an optimizer, and loss is calculated by categorical crossentropy, which is given by

(6) $$\begin{array}{r} loss=-\overset{N}{\underset{n=1}{\sum }}{ŷ}_{i1}logy_{i1}+{ŷ}_{i2}logy_{i2}+{ŷ}_{i3}logy_{i3}, \end{array}$$

where *N* is number of samples, *y*_*i*1_, *y*_*i*2_, *y*_*i*3_ are values of the label, which is also a form of one-hot code, and ŷ_*i*1_, ŷ_*i*2_, and ŷ_*i*3_ are three outputs from the output layer where *softmax* is used. Parameters in the model are updated by using back-propagation algorithm. The error between the desired output and the actual output is computed and the gradient descent method is applied to update parameters in order to minimize the error. Functions to update the weight and bias are shown by

(7) $$\begin{array}{r} W_{k}=W_{k}-\eta \frac{\partial E}{\partial W_{k}}, \end{array}$$

(8) $$\begin{array}{r} b_{k}=b_{k}-\eta \frac{\partial E}{\partial b_{k}}, \end{array}$$

where *W*_*k*_ is the weight matrix, *b*_*k*_ is the bias, and η represents the learning rate, *E* is the error. *E* is equal to *loss* in Equations (5) and (6). The results obtained from this CNN will be used as a benchmark for the performance comparison in section 3.

### 2.2. Sparse Autoencoder (SAE)

An autoencoder is a network including one input, one hidden, and one output layer, which is used to preserve the essence of the input data as much as possible and remove the potential noise in an unsupervised manner. The output data are therefore simplified, and important information from the input data are retained, which is beneficial for classification.

**Figure 2:**
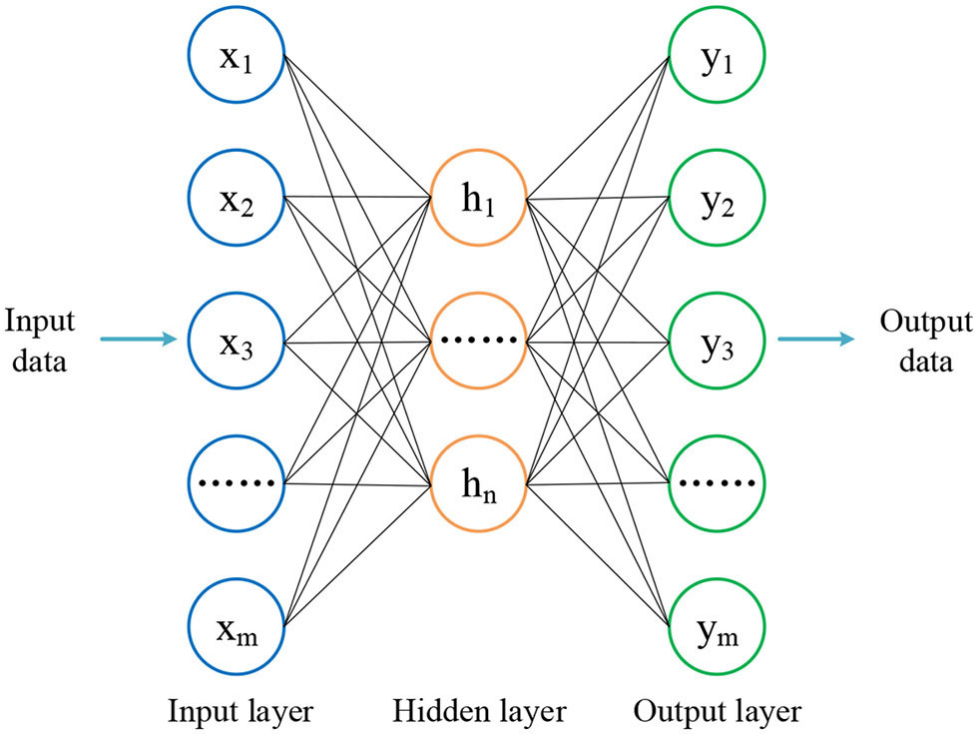
The autoencoder includes one input, one hidden, and one output layer.

The structure of autoencoder is shown in Figure 2. The whole data processing is divided into encoding and decoding phases. In the encoding phase, the dimension of input data are reduced in one layer. When the decoded data arrives at the hidden layer, the dimension of input data reaches the same as the number of neurons predefined for this layer. The encoding function of the hidden layer, *h*, is defined by

(9) $$\begin{array}{r} h=encoder\left(x\right)=f\left(W_{k}\times x+b_{k}\right), \end{array}$$

where $W_{k}\in R^{\;m\times n}$ is the weight matrix between input layer and the next layer. As defined previously in CNN, *b*_*k*_ is also the bias vector, and *f* represents the output function. The output function used in this part is *ReLU*, which is similar to the activation of the CNN. Differently from the encoding phase, in the decoding phase, the same number of neurons in output layers should be set as that of layers in encoding phase, in order to guarantee the output data has the same dimension as the input data. The decoding function is shown by

(10) $$\begin{array}{r} y=decoder\left(x\right)=g\left(W_{k}\times x+b_{k}\right), \end{array}$$

where $W_{k}\in R^{\;n\times m}$. After encoding and decoding phases, the model is trained, and the parameters could be obtained by minimizing the cost function, which is defined by

(11) $$\begin{array}{r} min\sum |E\left(x^{i},y^{i}\right)|, \end{array}$$

where *y*^*i*^ is output data and *x*^*i*^ is original input data. When the network is trained, output values are reconstructed, and the shape of which is equal to that of input data. Parameters of the model could be updated according to

(12) $$\begin{array}{r} W_{k}=W_{k}-\eta \frac{\partial E\left(x^{i},y^{i}\right)}{\partial W_{k}}, \end{array}$$

(13) $$\begin{array}{r} b_{k}=b_{k}-\eta \frac{\partial E\left(x^{i},y^{i}\right)}{\partial b_{k}}, \end{array}$$

where η denotes the learning rate of the network. *E* is an error in the SAE. For details of optimizer and *E*, they are the same as that in section 2.1 in binary-classification task.

In order to increase the generalization of the network and improve the training efficiency of the proposed network, a sparse constraint on the activity of the hidden representations is added in this work. Sparse constraint helps suppress activation of neurons in the hidden layer, and useful features can be extracted by autoencoder. Thus, the cost function in sparse autoencoder is described by

(14) $$\begin{array}{r} J_{sparse}\left(W,b\right)=J\left(W,b\right)+\beta \overset{m}{\underset{j=1}{\sum }}KL\left(\rho ||\hat{\rho_{j}}\right), \end{array}$$

where $\hat{\rho_{j}}$ is the average activation of hidden unit *j*, ρ is the sparsity level, and β is the weight of the sparsity penalty term. *KL* is the Kullback–Leibler divergence, which ensures the sparsity of neurons in hidden layer. *KL* is defined by

(15) $$\begin{array}{r} KL\left(\rho ||\hat{\rho_{j}}\right)=\rho \log\frac{\rho }{\hat{\rho_{j}}}+\left(1-\rho \right)\frac{1-\rho }{1-\hat{\rho_{j}}}, \end{array}$$

(16) $$\begin{array}{r} \hat{\rho_{j}}=\frac{1}{m}\overset{m}{\underset{i=1}{\sum }}f_{j}\left(x^{i}\right), \end{array}$$

where *m* denotes the number of samples at unite *j* in the hidden layer, and *f*_*j*_ denotes the activation of hidden neuron *j*.

### 2.3. Combined CNN-SAE-DNN

**Figure 3:**
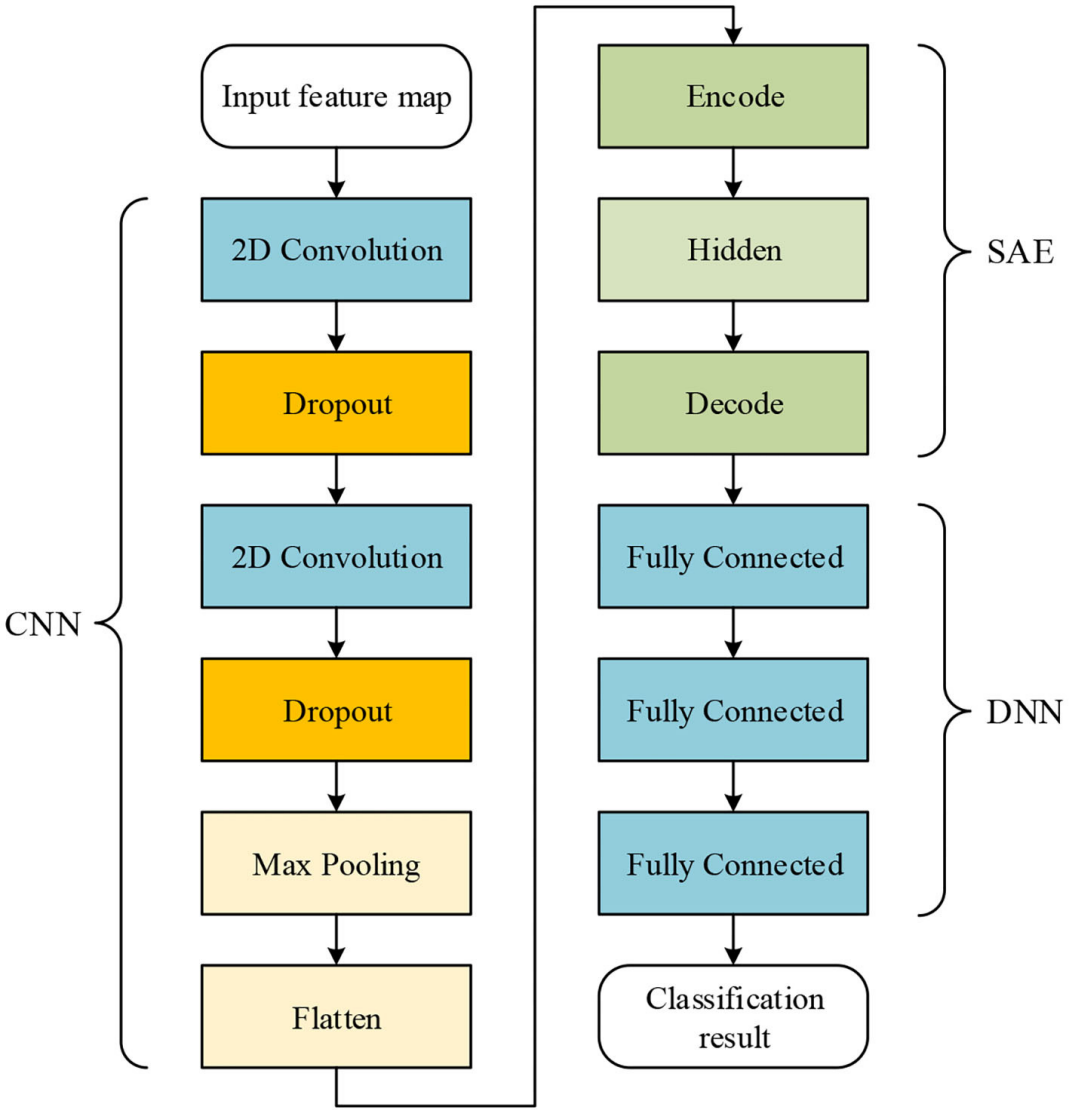
The proposed network includes the CNN, SAE, and DNN; the CNN and SAE are used for feature extraction, and the DNN is used for classification.

EEG signal is quite sensitive to a variety of factors during acquisition, such as environmental interference and the emotional fluctuations of humans. Therefore, EEG signals may be mixed with a variety of noise, which would undoubtedly influence the required brain patterns and the experimental results. In addition, in some experiments, subjects were unable to perform the emotion collection task successfully and the experimental results were deviated greatly. In order to overcome these problems, a deep learning network structure is proposed in this work. The structure of the proposed network is shown in Figure 3.

As shown by Figure 3, in the proposed network, the CNN structure consists of two convolutional layers and one max-pooling layer. Dropout connects to each convolutional layer. The SAE consists of one encode, one hidden, and one decode layer. In the DNN, there are three fully connected layers used for classification. Given features, such as PCC for input of the proposed network, the output of max-pooling layer is used as the input for the SAE. Finally, the output of the SAE is used as the input of the DNN for classification.

The training procedure is that the CNN with one fully-connected output layer are trained for some epochs using all samples and all features, and the output layer is abandoned after training. Then, by sending features to input the trained CNN, the output of the max-pooling layer can be obtained. The output is flattened to one-dimension data, and it is set as the input of SAE. After unsupervised learning of the SAE, data are reconstructed. The reconstructed data are divided for training and testing in the DNN, i.e., the CNN and SAE are trained separately. Thus, before data are classified in the DNN, training in the CNN and SAE can be seen as a part of feature extraction. It should be noticed that the DNN used for finally classification is not the fully-connected output layer abandoned from the CNN in the first step. The DNN is never trained before output of the SAE is obtained as input data for the DNN.

Another CNN with the same parameters and structure as the whole proposed network is set as comparison in order to test the performance of the proposed network fairly. When adding more layers into this CNN, accuracy does not improve and leads to an overfitting problem. For experiments on this CNN, features are split directly into 80% for training and the rest for testing.

## 3. Datasets and Experiments

In this section, two datasets of DEAP (Koelstra et al., 2012) and SEED (Zheng and Lu, 2015) are used to evaluate the proposed network model. Data processing methods and experiment results are presented.

### 3.1. Emotional EEG Datasets

The DEAP dataset was collected from 32 subjects when they were watching 40 sets of 1-min music and video clips. The age of the subjects ranges between 19 and 37 years old, and half of them were males. During the 40 trials for each subject, various signals were recorded as 40-channel data, including EEG, electromyograms, breathing zone, plethysmographs, temperature, and so on (Koelstra et al., 2012). The EEG signal was recorded at 512 Hz. The data was segmented into trials of 60 s, and a bandpass frequency filter was applied after that. After each trial, the participants were asked to do a self-assessment about their emotional levels, including four different scales, such as valence, arousal, dominance, and liking.

The EEG signal is downsampled into 128 Hz for the experiments in this work, where the frequency of EEG data are from 4.0 to 45.0 Hz. Valence and arousal are the two scales chosen for this work. Each of them ranges from one (low) to nine (high), and scales are divided into two parts to construct our binary-classification tasks. Similarly to the work in Koelstra et al. (2012), valence is divided into high (ranging from five to nine) and low valence (range from one to five) according to the valence scale, and according to the arousal scale, arousal is divided into high (ranging from five to nine) and low arousal (ranging from one to five).

The SEED dataset was collected from 15 subjects (seven males) when they were asked to watch 15 film clips. The duration of each film clip was about 4 min, and each film as easily understood in order to elicit emotion of 15 subjects participating in the experiments effectively. There were 15 trials for each subject and each trial lasted for 305 s consisting of a hint of start for 5 s, a movie clip for 4 min, a self-assessment for 45 s, and a rest for 15 s. EEG data in SEED dataset was collected from 62 electrodes, which includes more information than the DEAP dataset. After collection, EEG data was downsampled to 200 Hz and applied with a bandpass filter from 0 to 75 Hz.

Similar to the DEAP dataset, in this dataset, the data are applied with a frequency filter from 4.0 to 45.0 Hz in order to equitably evaluate the proposed network. Negative, positive, and neutral are emotion labels in this dataset that represent the subjects' emotion states during each experiment. Label value of negative, positive and neutral is −1, 1, and 0, respectively. Thus, labels in the SEED dataset include three categories.

### 3.2. Experiment Setting

In order to test the efficacy of the proposed network, the CNN model and the proposed network are trained by using data obtained from two time windows of different lengths; in total, four groups of experiments were conducted. For experiments in the CNN used for comparison, after feature extraction of EEG data, 80% samples are used as training data and the rest samples are used as test data among all of the data. Average accuracy is calculated from accuracies of the last 10 epochs in each experiment. For the proposed network, before training data and testing data were divided, the CNN and SAE in the proposed network were trained using features. After that, features are sent to the input of the CNN, and the output data of SAE is obtained. The output data after feature extraction were divided into 80% for training and 20% for testing in the DNN. In this work, Keras and Tensorflow (Abadi et al., 2016) ere used for the proposed network implementation. For detailed free parameters in the proposed network, they are described in sections 3.3 and 3.4, respectively.

### 3.3. Experiments on the DEAP Dataset

Length of data in the DEAP dataset is 63 s, and the first 3 s are removed in the experiments. Then band pass filtering is then applied. Among 40 channels, EEG data are contained in 32 channels, which are chosen for experiments. After that, EEG signals are decomposed into α (1–7 Hz), β (8–13 Hz), θ (14–30 Hz), and γ bands (30–45 Hz). After band pass filtering, signal windowing on four frequency bands is applied. EEG signals are divided into short time frames in order to facilitate signal processing, thus time windows with different overlaps are applied to EEG data in order to increase samples for training. Two window sizes, 8 and 12 s, are used for evaluating the proposed network. From the start of each recorded EEG signal, data are segmented by a sliding time window with an overlap for each frequency band. For each trial of 60 s, 14 segments are obtained using an 8-s time window moving every 4 s, and seven segments are obtained using a 12-s time window moving every 8 s. Finally, from a total of 32 participants, 17,920 (14 segments × 40 trials × 32 participants) and 8,960 (seven segments × 40 trials × 32 participants) samples are obtained using time windows of 8 and 12 s, respectively. Segment labels are the same as the label of the original sample.

**Figure 4:**
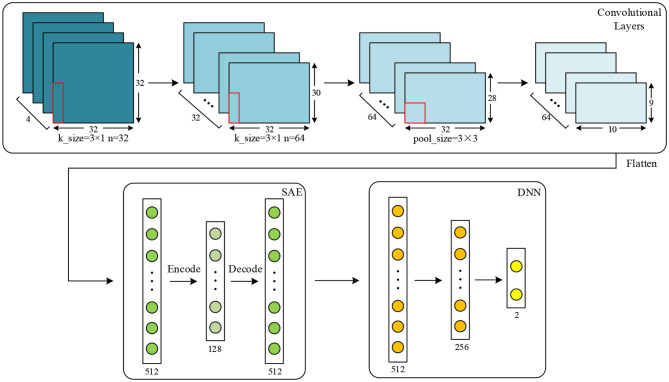
Configuration of the proposed network for the DEAP dataset.

After that, three different features, namely PCC, PCA, and SC, are extracted to evaluate the proposed network. For PCC-based features, PCC of data in every two channels are calculated, and a 32 × 32 PCC matrix is constructed for one sample. For PCA-based features, dimension of data from each channel is reduced into 32, and features with the shape of 32 × 32 are obtained. For SC-based features, four different characteristics are extracted, including variance, mean, kurtosis, and skewness. These statistical characteristics of data are calculated together, and a 32 × 4 matrix is finally obtained. In the proposed work, the features are separately extracted in each of the frequency bands (α, β, θ, and γ bands). According to the work in Wang et al. (2018) and other similar researches, data of four frequency bands are used together in order to get the best results. After data are processed, for the data obtained using a time window of 8 s, the shapes of the above three different feature matrixes are 17,920 × 4 × 32 × 32, 17,920 × 4 × 32 × 32, and 17,920 × 4 × 32 × 4, respectively. For data obtained using a time window of 12 s, they are 8,960 × 4 × 32 × 32, 8,960 × 4 × 32 × 32, and 8,960 × 4 × 32 × 4, respectively. Detailed configuration of the proposed network for DEAP dataset is shown by Figure 4. For SC, input shape is 32 × 4. These features are two-dimensional, which are suitable inputs for the CNN and the proposed network.

As shown in Figure 4, for the DEAP dataset, two convolutional layers and one max-pooling layer are applied for the proposed network. Kernel size is set to 3 × 1, and pooling size is set to 3 × 3. The input data shape is 32 × 32. The numbers of kernels in convolutional layer are set to 32 and 64, respectively. In the SAE, the numbers of neurons in encode, hidden, and decode layers are set to 512, 128, and 512, respectively. In the DNN, the numbers of three fully connected layers are set to 512, 256, and 2, respectively. In the proposed network, the training epochs, batch size, and learning rate in the CNN are set to 50, 128, and 0.01. Epoch, batch size, and learning rate in the SAE are set to 100, 64, and 0.01, respectively. For those of the DNN, they are set to 100, 128, and 0.01, respectively.

In the proposed network, the training epochs are carried out in convolutional layers, and the SAE for features extraction, training, and testing epochs are carried out in the DNN for classification. Another CNN with the same parameters and structure as the proposed network served as a baseline method to evaluate the performance of the proposed network. The epoch, batch size, and learning rate of this CNN were set to 100, 128, and 0.01. Parameters in this CNN were the same as that of the proposed network. The data results of the experiments using a time window of 8 s are shown by Table 1.

**Table 1:** Average accuracies comparisons of the DEAP dataset using different features extracted from the data with a length of 8 s between two networks.

**Network**	**Labels**	**PCC (%)**	**PCA (%)**	**SC (%)**
CNN	Valence Arousal	78.80 82.25	73.32 72.76	71.10 73.04
Proposed network	Valence Arousal	89.49 92.86	75.59 85.87	81.93 82.94

From Table 1, among all features extracted from EEG data, we can see the PCC feature was demonstrated to be better than most of the other features on both the CNN and the proposed network. The proposed network can reach a recognition accuracy of 92.86% on arousal by using PCC. Moreover, recognition accuracies of most experiments on the proposed network are better than the CNN (3.27–13.11% improvement). As described previously, this is due to the inclusion of SAE, which can not only reconstruct data from convolutional layers and pooling layer but can also extract features further and make the data easier to be recognized than the CNN.

**Figure 5:**
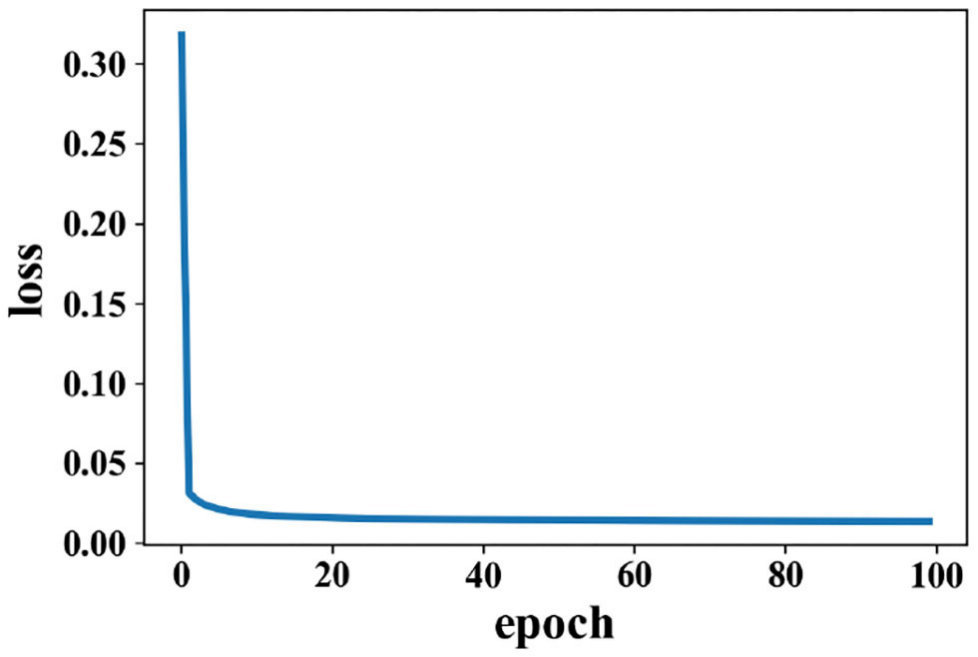
Change of loss when data are reconstructed in the SAE on the DEAP dataset.

Training for loss of SAE is shown in Figure 5; data reconstruction is achieved when the loss does not change sharply, and data reconstruction is fast during the training process of SAE. For other extracted features (except PCC), the recognition accuracy of each method is better than the work in Zhang et al. (2016a) (81.21% for valence and 81.26% for arousal).

**Figure 6:**
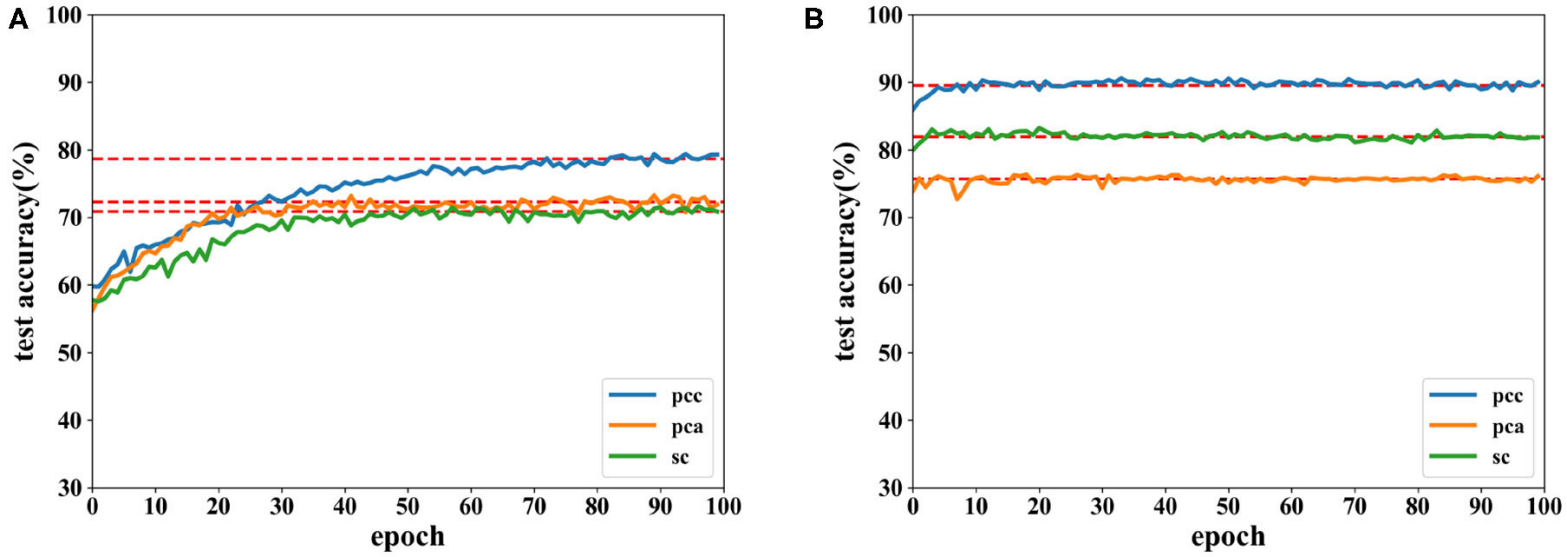
Accuracy comparison of two networks on valence using data with a length of 8 s on the DEAP dataset in which **(A)** is result of the CNN and **(B)** the result of the proposed network.

**Figure 7:**
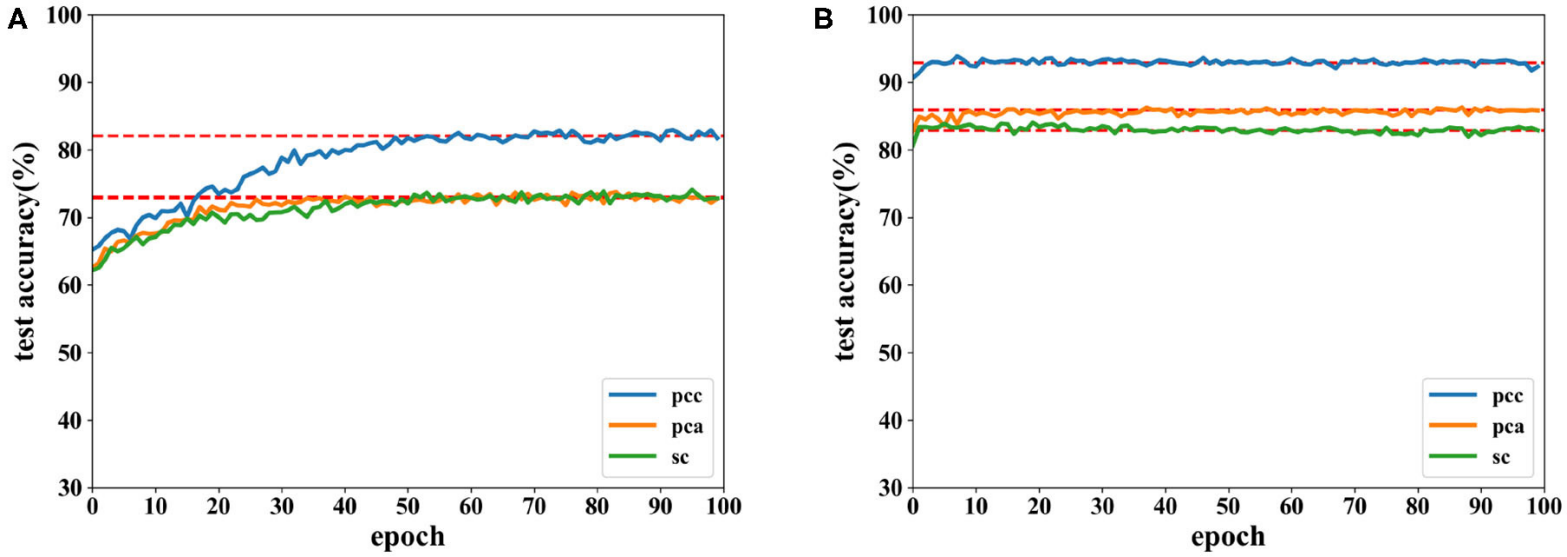
Accuracy comparison of two networks on arousal using data with a length of 8 s on the DEAP dataset in which **(A)** is result of the CNN and **(B)** the result of the proposed network.

Figures 6, 7 show the accuracies of the CNN and the proposed network. Red lines in figures denote the average accuracy of the last 10 epochs. It can be seen that the accuracy of the CNN gradually converges. For the proposed network, the accuracy converges rapidly at the beginning of the epoch after fewer than 10 epochs. This is because features are easy to recognize using output data obtained from the SAE before they are classified by the DNN. For features extracted by the PCC and other methods, the accuracy of a proposed network has a faster convergence than CNN.

Similarly, results using data obtained from a time window of 12 s are shown in Table 2. From Table 2, we can see that the accuracy obtained using data with a length of 12 s is lower than that of 8 s. It is more difficult to collect emotion information when the stimulation time is increasing. Most studies related to the classification of EEG data was focused on a short length of time. In this experiment, higher classification accuracy is achieved on data of 12 s than that of shorter length in other studies; this is like the work in Zhang et al. (2017), which exhibits the effectiveness of the proposed network.

**Table 2:** Average accuracy comparisons on the DEAP dataset using different features extracted from data with a length of 12 s between two networks.

**Network**	**Labels**	**PCC (%)**	**PCA (%)**	**SC (%)**
CNN	Valence Arousal	75.13 76.12	67.23 69.20	66.09 69.48
Proposed network	Valence Arousal	82.16 85.47	76.34 79.11	73.41 75.44

**Figure 8:**
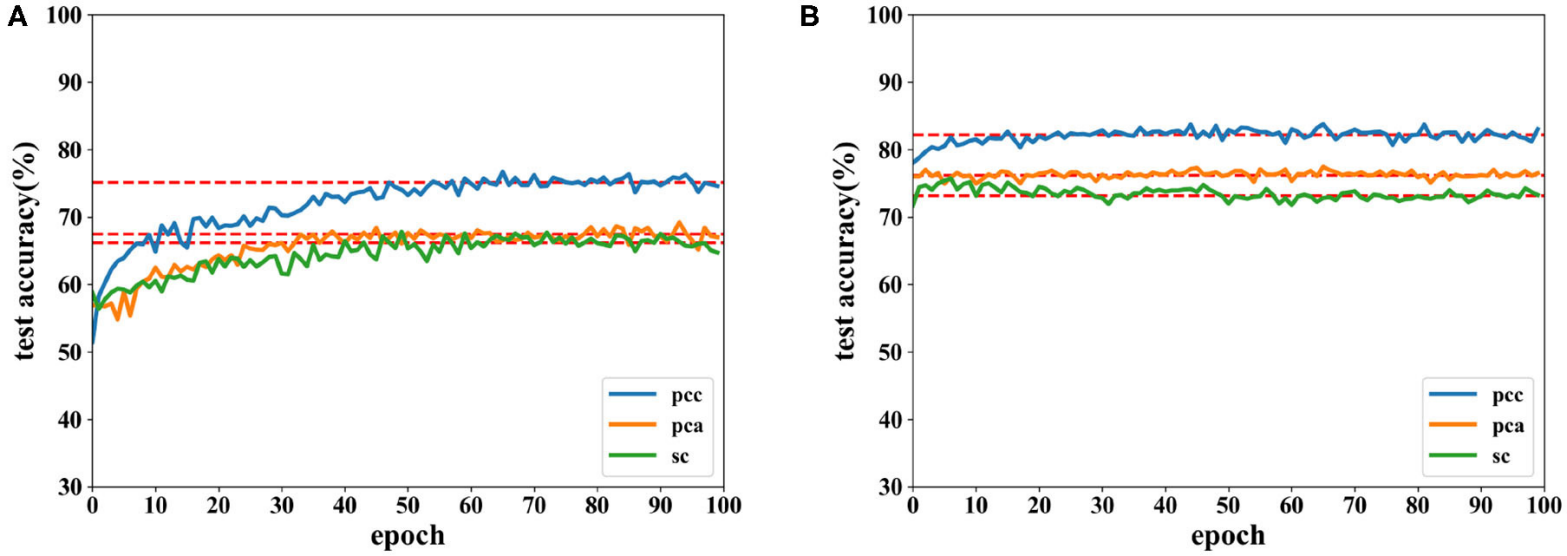
Accuracy comparison of two networks on valence using data with a length of 12 s on DEAP dataset in which **(A)** is the result of the CNN and **(B)** the result of the proposed network.

**Figure 9:**
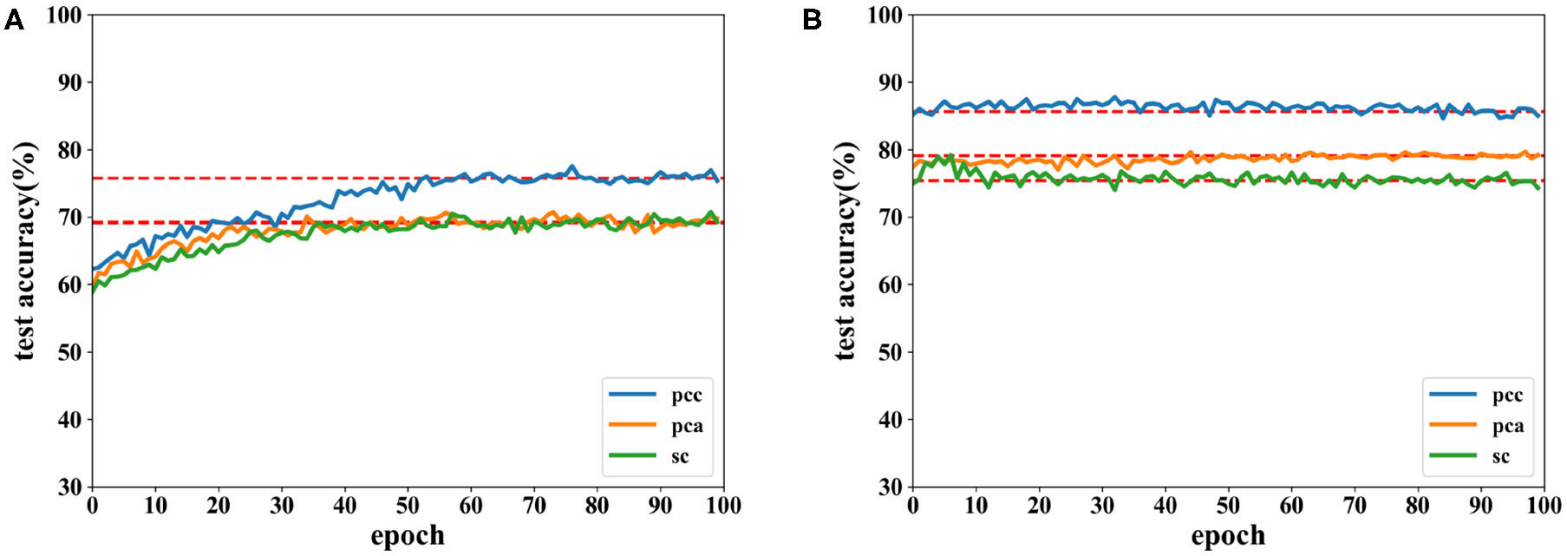
Accuracy comparison of two networks on arousal using data with a length of 12 s on the DEAP dataset in which **(A)** is the result of the CNN and **(B)** the result of the proposed network.

Accuracies for classification on data of 12 s on both CNN and the proposed network are shown by Figures 8, 9. It can be found that higher recognition accuracy is obtained by the proposed network. Moreover, the classification accuracy of the proposed network has a faster convergence in each experiment.

The results in this subsection demonstrate that accuracies can reach 92.86% for data of 8 s and 85.47% for data of 12 s. When the same feature is used for comparison, the proposed network is more powerful in classifying the EEG emotion data than the CNN. Finally, the proposed network has a quicker convergence speed.

### 3.4. Experiments on SEED Dataset

There are a total of 675 trials in the SEED dataset. According to the work in Zheng and Lu (2015), the first sample of each subject was chosen, and a total of 225 samples were then obtained. Due to the different data length of each channel, the 80 s data segment was chosen to reduce the influence of unstable signals at the beginning and end of the whole signal; finally, data with the shape of 16,000 × 225 × 62 were obtained. Moreover, the data were processed as the same way as in the DEAP dataset: each sample was divided into different frames with different time windows. Two time windows, 8 and 12 s, were also used in the SEED dataset. A total of 19 and nine segments were obtained separately from data using a time window of 8 s moving every 4 s and a time window of 12 s moving every 8 s for each sample, respectively. Thus, in a total of 225 trials, 4,275 (19 segments × 15 trials × 15 participants) and 2,025 (9 segments × 15 trials × 15 participants) samples were obtained, respectively.

**Figure 10:**
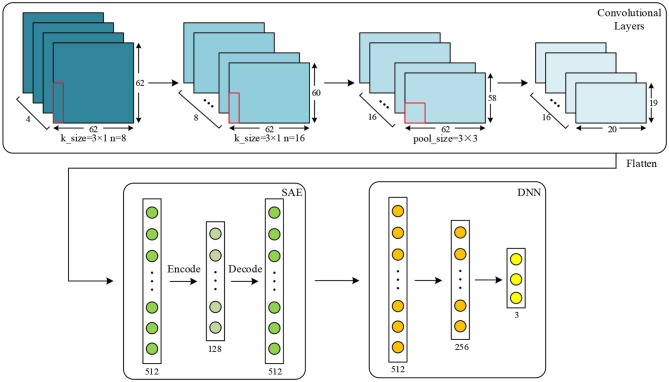
Configuration of the proposed network for the SEED dataset.

The detailed configuration of the proposed network for the SEED dataset is shown in Figure 10. The amount of data extracted from this dataset is much less than from the DEAP dataset, and the two classifiers used on this dataset are thus a little different. For PCC, input data are 62 × 62, and the numbers of kernels are separately set to eight and 16 in two convolutional layers. In the SAE and DNN, the number of each layer is set the same as that on the DEAP dataset except that the number of the output layer is set to three because this is a three-classification task on a SEED dataset. After training in the CNN and SAE for feature extraction, the DNN is used for the final classification. Similarly to the DEAP dataset, the same features are extracted for the SEED dataset. For PCA and SC, the input shape is 62 × 62 and 62 × 4, respectively. For the CNN used for comparison, parameters are also set as the same as the proposed network.

**Figure 11:**
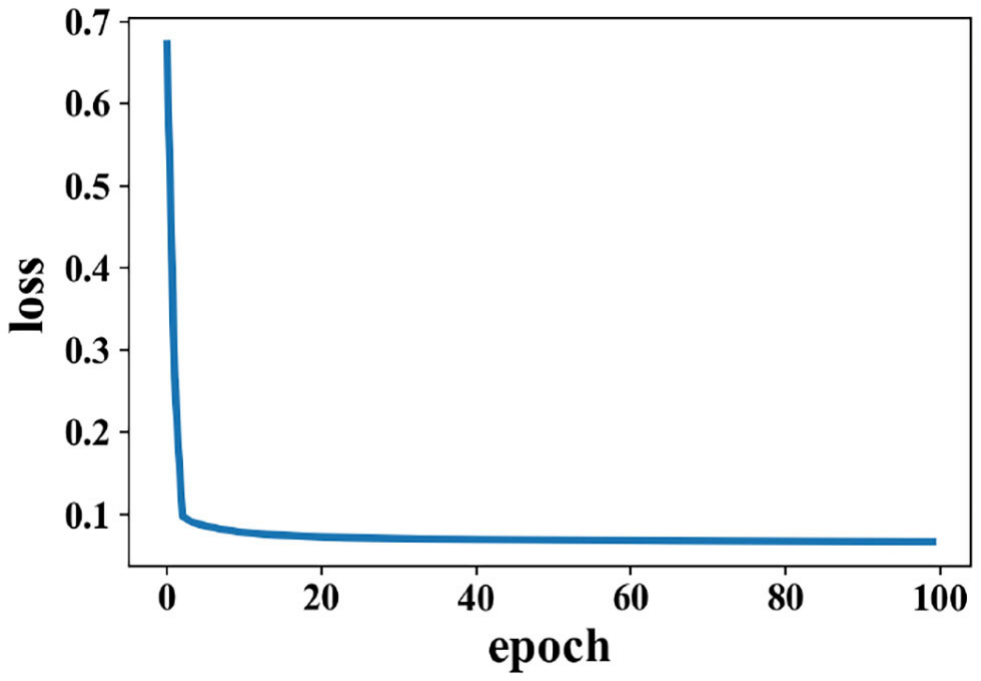
Change of loss when data are reconstructed by the SAE on the SEED dataset.

The experiment results of data obtained from time windows of 8 and 12 s are shown in Table 3. The accuracy under this dataset is higher than the DEAP dataset. The highest average accuracy could reach 96.77%, which is better than the work in Wang et al. (2018), 90.2%. For the data obtained from time window of 12 s, the best accuracy could reach 94.62%, which shows that PCC-based features exhibit a better performance than others. The reconstruction of data by the SAE due to the change in loss on the SEED dataset is shown by Figure 11. Loss drops immediately following several epochs, i.e., the data reconstruction can be achieved quickly when the SAE is being trained.

**Table 3:** Average accuracy comparisons on SEED dataset using different features extracted from data with a length of 8 s between two networks.

**Network**	**Data length (s)**	**PCC (%)**	**PCA (%)**	**SC (%)**
CNN	8 12	93.71 91.53	77.66 70.59	83.32 75.48
Proposed network	8 12	96.77 94.62	88.90 70.62	87.73 79.09

**Figure 12:**
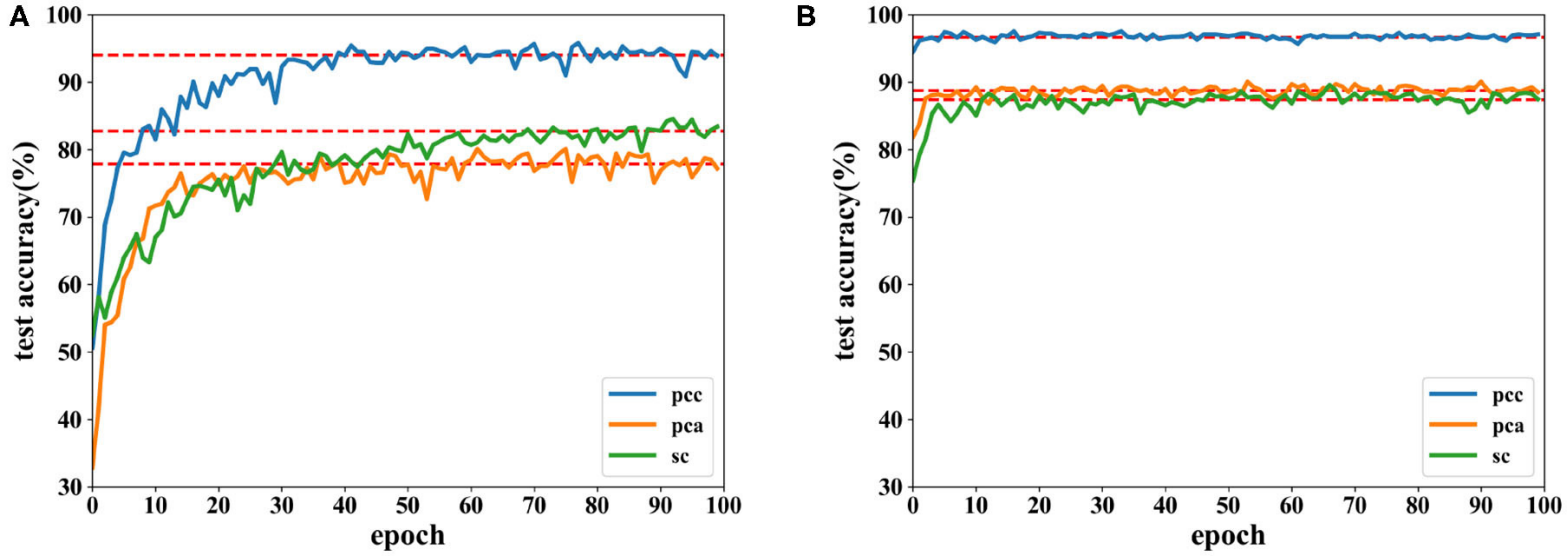
Accuracy comparison of two networks using data with length of 8 s on the SEED dataset in which **(A)** is the result of the CNN and **(B)** the result of the proposed network.

**Figure 13:**
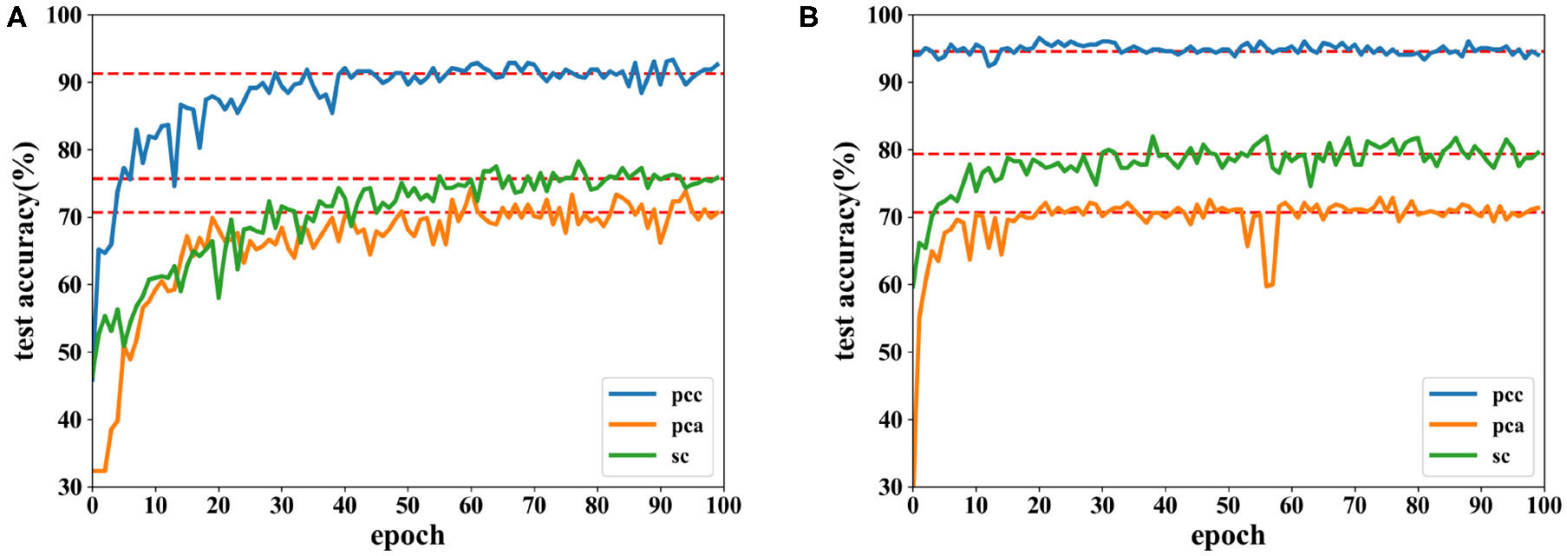
Accuracy comparison of two networks using data with a length of 12 s on the SEED dataset in which **(A)** is the result of the CNN and **(B)** the result of the proposed network.

Accuracies under different features extracted from data of 8 and 12 s are depicted in Figures 12, 13. It is shown that recognition accuracies of the proposed network are better than the CNN for almost all features, especially the PCC-based features. The proposed network can achieve faster convergence on classification accuracy than the CNN on the SEED dataset. Experiments on these two datasets shows that the proposed network performs better than original the CNN in emotion recognition.

**Figure 14:**
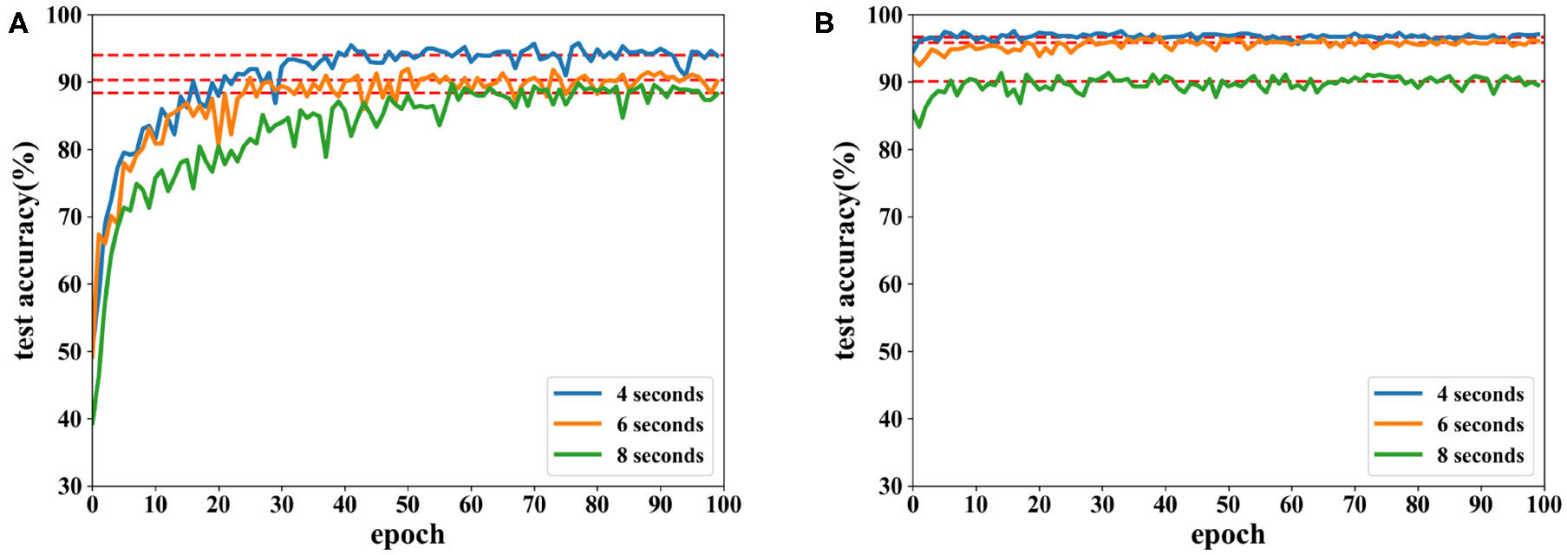
Accuracy comparison of two networks using features extracted from different lengths of overlap on the SEED dataset in which **(A)** is the result of the CNN and **(B)** the result of the proposed network.

Moreover, EEG data divided by a fixed time window with different overlaps on the SEED dataset are tested. Besides a time window of 8 s with an overlap of 4 s, overlaps of 6 and 8 s are also tested. Due to the highest accuracy, PCC-based features are used in these experiments, and classification results are displayed in Figure 14.

As seen in Figure 14, recognition accuracy could reach the highest value while the overlap is 4 s. The shorter the overlap is, the more similar the neighboring data segments are, i.e., features could be learned better when similar information is included in each trial. However, when the overlap is too short, the number of data segments increases, which requires longer time for training. In this experiment, data with a length of 8 s and overlap of 4 s could achieve the best result.

In a short summary, the best recognition could reach 96.77% on the three-class classification. The proposed network is demonstrates to be more powerful in classifying EEG emotion data than the CNN on the SEED dataset. For the data with the same length, length of overlap has an impact on recognition accuracy where 4-s overlap obtained the best performance. In addition to this, the proposed network is also compared with other research works using the DEAP and SEED datasets, and the results can be seen in Table 4. For complexity analysis, the number of parameters are 7.55 × 10^5^ and 7.50 × 10^5^ for the networks used for DEAP and SEED, respectively.

**Table 4:** Performance comparisons with other approaches.

**Classification methods**	**DEAP dataset**		**SEED dataset (%)**
	**Valence (%)**	**Arousal (%)**	
CNN + statistical methods (Tripathi et al., 2017)	81.4	73.4	/
Gaussian Bayes (Koelstra et al., 2012)	57.6	62.0	/
Deep SAE + RSP (Zhang et al., 2017)	73.1	80.8	/
BDGLS + DE (Wang et al., 2018)	/	/	93.7
DGCNN + DE (Zhang et al., 2016)	/	/	90.4
GP + LVM (García et al., 2016)	88.3	90.6	/
BLSTM + DE (Wang et al., 2019)	/	/	94.96
Physe Space Dynamics (Soroush et al., 2019)	84.6	87.4	/
SDEL + PCA (Ullah et al., 2019)	82.8	74.5	/
**This work [PCC]**	89.49	92.86	96.77

Table 4 shows the results of García et al. (2016) achieved 88.3% on valence and 90.6% on arousal. However, data used for experiments are limited, and the classification model is a better fit for classifying small amounts of data with high dimensions. The approach of Koelstra et al. (2012) used a Gaussian Bayes classifier, and experiment results proved that EEG signals are effective in emotion recognition of the DEAP dataset. The recent study (Tripathi et al., 2017) used extracted data for classification, and better accuracy results were obtained by using the CNN, where the classification accuracy of valence and arousal is 81.4 and 73.4%, respectively. The approaches of García et al. (2016) and Wang et al. (2018) used DE-based features and dynamical graph convolutional neural networks, and the accuracy achieved 93.7%. In the approach of Wang et al. (2019), BLSTM and other machine learning classifiers, such as SVM and LR were used for emotion recognition. BLSTM achieved the best accuracy of 94.96% on the SEED dataset, which is better than SVM and LR. In the approach of Soroush et al. (2019), phase space dynamics were introduced to classify emotions, achieving 87.42% on arousal and 84.59% on valence, respectively. A sparse discriminative ensemble was used for feature extraction in Ullah et al. (2019) and achieved 82.81% on valence and 74.53% on arousal, respectively. In this work, both the DEAP and SEED datasets are used for experiments, where accuracies achieve 89.49% and 92.86% in valence and arousal on the DEAP dataset, respectively, and 96.77% on the SEED dataset. Results demonstrate that the proposed network is more powerful than the CNN and other approaches.

## 4. Discussion and Conclusion

There are some points worth discussing. First, the proposed model can be trained using an end-to-end method, which is different from this work. The end-to-end training method was tested, and it obtained a similar performance. However, the training model can be further investigated and optimized in a future work. Second, labels are used in feature extraction. It should be noted that many feature extraction algorithms use labels such Relief and ReliefF (Kira and Rendell, 1992), where feature weights are calculated according to samples in the same and different classes. Label information has been used in the feature extraction process (Bohgaki et al., 2014; Zhang et al., 2016b). Third, constructing an autoencoder-like structure is another method of emotion recognition, and this can be investigated in a future work.

In this work, a new deep network is proposed to classify EEG signals for emotion recognition. The CNN and the proposed network are applied for two different datasets, i.e., the DEAP and SEED datasets. In the proposed network, the CNN and SAE are trained for feature extraction in which, by combining supervised learning of the CNN and unsupervised learning of the SAE, more useful features are extracted. Experimental results show that the proposed network achieves a better performance than the CNN and other approaches. It also shows that when embedding an SAE structure into a CNN, the accuracy is better compared to a CNN with the same parameters and structure as the proposed network. In the proposed network, three different features are extracted for classifications. Results showed that, by using PCC-based features, the average recognition accuracy of the proposed network can reach 89.49% on valence and 92.86% on arousal for DEAP and 96.77% for SEED, where the proposed network has a faster convergence speed. In addition, overlap length also affects the performance, and results under the SEED dataset showed that data of 8 s with an overlap of 4 s can achieve the best result. It is also found that the data processed by the SAE is easily classified in the proposed network, which indicates that the SAE is effective in extracting features from EEG data. Future works will consider using the SAE and other classifiers to further improve the classification performance.

## Data Availability Statement

All datasets presented in this study are included in the article/supplementary material.

## Author Contributions

JL, GW, SQ, and YL developed, implemented, and evaluated the neural network algorithm. JL and GW wrote and revised the manuscript. SY, WL, and YB analyzed the performance of the proposed network and reviewed the manuscript. All authors contributed to the article and approved the submitted version.

## Conflict of Interest

The authors declare that the research was conducted in the absence of any commercial or financial relationships that could be construed as a potential conflict of interest.
